# Molecular Characterization and In Silico Analyses of Maurolipin Structure as a Secretory Phospholipase *A*_2_ (sPLA_2_) from Venom Glands of Iranian *Scorpio maurus* (Arachnida: Scorpionida)

**DOI:** 10.1155/2022/1839946

**Published:** 2022-10-03

**Authors:** Parisa Soltan-Alinejad, Hamzeh Alipour, Aboozar Soltani, Qasem Asgari, Amin Ramezani, Davood Mehrabani, Kourosh Azizi

**Affiliations:** ^1^Student Research Committee, Department of Medical Entomology, School of Health, Shiraz University of Medical Sciences, Shiraz, Iran; ^2^Research Center for Health Sciences, Institute of Health, Department of Medical Entomology, School of Health, Shiraz University of Medical Sciences, Shiraz, Iran; ^3^Department of Parasitology and Mycology, School of Medicine, Shiraz University of Medical Sciences, Shiraz, Iran; ^4^Shiraz Institute for Cancer Research, School of Medicine, Shiraz University of Medical Science, Shiraz, Iran; ^5^Department of Medical Biotechnology, School of Advanced Medical Sciences and Technologies, Shiraz University of Medical Sciences, Shiraz, Iran; ^6^Li Ka Shing Center for Health Research and Innovation, University of Alberta, Edmonton, AB, Canada; ^7^Stem Cell Technology Research Center, Shiraz University of Medical Sciences, Shiraz, Iran

## Abstract

The venom is a mixture of various compounds with specific biological activities, such as the phospholipase  *A*_2_ (PLA_2_) enzyme present in scorpion venom. PLA_2_ plays a key role in inhibiting ryanodine receptor channels and has neurotoxic activity. This study is the first investigation of molecular characterization, cloning, and in silico analyses of PLA_2 _ from Iranian *Scorpio maurus,* named Maurolipin. After RNA extraction from *S. maurus* venom glands, cDNA was synthesized and amplified through RT-PCR using specific primers. Amplified Maurolipin was cloned in TA cloning vector, pTG19. For in silico analyses, the characterized gene was analyzed utilizing different software. Maurolipin coding gene with 432 base pair nucleotide length encoded a protein of 144 amino acid residues and 16.34 kilodaltons. Comparing the coding sequence of Maurolipin with other characterized PLA_2 _ from different species of scorpions showed that this protein was a member of the PLA_2 _ superfamily. According to SWISS-MODEL prediction, Maurolipin had 38.83% identity with bee venom PLA_2 _ with 100% confidence and 39% identity with insect phospholipase *A*_2_ family, which Phyre2 predicted. According to the three-dimensional structure prediction, Maurolipin with five disulfide bonds has a very high similarity to the structure of PLA_2_ that belonged to the group III subfamily. The in silico analyses showed that phospholipase *A*_2_ coding gene and protein structure is different based on scorpion species and geographical condition in which they live.

## 1. Background

The order of scorpions is the oldest one of venomous arthropods living over 400 million years ago [1, 2]. So far, over 2360 scorpion species have been identified and classified as 19 families [3]. The scorpion species have venom glands used to secrete venom into venom sacs to defend and capture the prey [4]. There are various scorpion species, which the stings cause respiratory dysfunctions, cardiotoxicity, and neurotoxicity [5]. The secreted compounds from the scorpion venom gland are a complex mixture of proteins, nonproteins, and some unknown compounds with unknown functions, including inorganic salts, mucoproteins, carbohydrates, peptides, lipids, free amino acids, nucleotides, water, and enzymes such as phospholipase *A*_2_ (PLA_2_) [6–8]. PLA_2_ plays a vital role in inhibiting ryanodine receptor channels and has neurotoxic activity [9].

PLA_2_ is an enzyme that hydrolyzes glycerophospholipids at the position of sn‐2 of phospholipids and releases free fatty acids (oleic acid (OA) and arachidonic acid (AA)) and lysophospholipids [10, 11]. This superfamily is classified into 15 groups and many subgroups [12]. PLA_2 _ superfamily has six principal types, including cytosolic PLA_2_ (cPLA_2_), platelet-activating factor cetyl hydrolases (PAF-AH), Ca^2+^-independent PLA_2_ (iPLA_2_), lysosomal PLA_2_, adipose-specific PLA_2_ (AdPLA_2_), and secreted phospholipase *A*_2_ (sPLA_2_) [12–14].

sPLA_2_ as a calcium-dependent enzyme is a typical enzyme in the venom of a snake, lizard, bee, and scorpion that has been identified till now [10, 15–17]. This low-molecular weight enzyme is 13-15 kilodaltons (kDa) [18]. So far, 17 isoforms of sPLA_2 _ have been identified and divided into groups I-III, V, and IX-XIV [19]. sPLA_2 _ from the venom glands of scorpions is classified as a group III subfamily [20]. sPLA_2 _ has been identified from different species of scorpions till now, including *Pandinus imperator* [21, 22], *Hemiscorpius lepturus* [23–25], *Anuroctonus phaiodactylus* [20], *Heterometrus fulvipes* [26], *Heterometrus laoticus* [10], and Tunisian *Scorpio maurus* [27].

As a country located in the Middle East, due to its climate, Iran has a good potential for scorpions' life [28]. According to previous studies, Iran has one of the highest ranks in terms of scorpion envenomation over the world [29]. The last research has shown that Iran possesses 68 scorpion species that comprise 19 genera and four families [30]. The data estimated that the south and southwest of Iran possess about 95% of scorpion species, so were known as densely populated areas [31]. Fars Province, located in the southwest of Iran, has hot and humid weather and scorpions are important public health problem in this region [32]. *Scorpio maurus,* as a species of the Scorpionidae family, is a notable species in this province [33].

## 2. Objectives

This study aimed to identify PLA_2_ from the venom glands of Iranian scorpion, *S. maurus,* based on molecular characterization and in silico analyses.

## 3. Methods

### 3.1. Scorpion Collection

**Figure 1:**
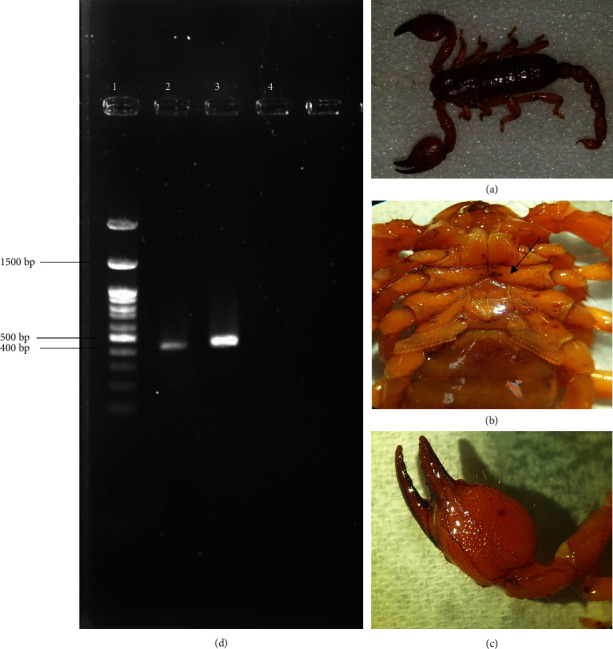
Morphological characteristics of the *Scorpio maurus* investigated in the current study. The general morphology of *S.maurus* (a), the pentagonal-shaped sternum of the Scorpionidae family (b), and short and bulky pedipalps (c). Amplification of Maurolipin (Iranian *S. maurus*PLA_2_) through RT-PCR using specific primers (d); Lane 1 is ladder 100 (bp) DNA marker, Lanes 2 and 3 are PLA_2_ (432 bp), and Lane 4 is negative control.

The scorpions were collected from Fars Province, Zarrin Dasht County, in the southwest of Iran and transferred alive to the laboratory of Medical Entomology in Shiraz University of Medical Sciences, Shiraz, Iran. The morphological characteristics of this species are shown in Figures 1(a)-1(c). Samples were identified via a valid key [30]. Before RNA extraction, the venom of the collected scorpions was milked manually to release the venom. Three days after venom milking, the telson of one scorpion was separated and stored at −70°C. Other parts of the body were stored at ethanol 96% and were kept in the archives of the Museum of the Department of Medical Entomology in Shiraz University of Medical Sciences.

### 3.2. Primer Designing

To design the gene-specific primers (GSPs), researchers obtained the mRNA sequences of PLA_2_ from different species of scorpions such as Tunisian *S. maurus* (GenBank : MF347455), *H. lepturus* (GenBank : KX924472), *A. phaiodactylus* (GenBank : AY571967.1), and *Opisthacanthus cayaporum* (GenBank : FM998793.1) from the National Center for Biotechnology Information (NCBI). The standard size band was 432 base pair (bp). The specific primers were 5′-TCCAAAGAAGAAATGGA (forward primer) and 5′-GTCTTTGTAGCTCTTTTTCCAGG (reverse primer).

### 3.3. RNA Extraction

Total RNA was extracted from the venom glands of one *S. maurus* telson by using a High Pure RNA Isolation Kit, Roche®. The RNA sample was treated enzymatically by DNase based on the manufacturer's manual. Extracted RNA concentration was measured using a Nanodrop (Analytik Jena®).

### 3.4. Reverse Transcription Polymerase Chain Reaction (RT-PCR)

According to the manufacturer's instruction, 3 *μ*L of total RNA was used as a template for cDNA synthesis by AccuPower® CycleScript RT Premix with (*dN*_6_) (Bioneer Company, Korea). 0.1 to 1 *μ*g of RNA template was filled up to the 20 *μ*L volume with sterile water and was dissolved by vortexing. cDNA synthesis reaction was performed in four steps according to the manufacturer's manual, including 30 sec at 25°C for primer annealing, 4 min at 45°C for cDNA synthesis, 30 sec at 55°C for melting secondary structure and cDNA synthesis, and 5 min at 95°C for heat inactivation. The synthesized cDNA was kept at −70°C for amplification steps as a template. Researchers amplified the desired DNA sequences in total 20 *μ*L volume containing 10 *μ*L Taq DNA Polymerase Master Mix RED (2X), 1 *μ*L of forward primer, 1 *μ*L of reverse primer, 1 *μ*L of synthesized cDNA, and finally 7 *μ*L of sterile water. It was performed to 35 cycles of 30 sec at 94°C as denaturation, 30 sec at 50°C as annealing temperature, 30 sec at 72°C as an extension, and 10 min at 72°C for a final extension.

### 3.5. TA Cloning

PCR products were run onto 1% tris borate EDTA (TBE) agarose gel with an appropriate DNA ladder (100 bp). Our specific band was observed in the gel documentation instrument, and the selected band was purified according to the protocol of GeneAll® kit.

The purified PCR product was ligated to the linearized pTG19 vector with a TA cloning kit (Vivantis®) according to the manufacturer's instructions in 1 : 3 ratio. *Escherichia coli* strain DH5*α* competent cells were prepared before ligation reaction and stored at −70°C. Ligation mixtures were transformed in prepared competent cells by heat shock method. Blue-white screening technique was used on Luria-Bertani (LB) agar plates containing ampicillin (100 *μ*g/ml), 5-bromo-4-chloro-3-indolyl-*β*-D-galactopyranoside (X-Gal) (40 *μ*g/ml), and isopropyl *ß*-D-1-thiogalactopyranoside (IPTG) (40 *μ*g/ml) for detection of recombinant colonies . Each white colony was suspended in 30 *μ*l of sterile water and boiled for 10 min. 1 *μ*l of that has been used as a DNA template in PCR reaction for the characterization of recombinant colonies containing target insert using specific and universal M13 primers. After detecting the recombinant colonies based on PCR technique, plasmid DNA was extracted using the GeneAll® plasmid isolation kit according to manufacturer's manual. Maurolipin coding gene was sequenced using universal M13 forward and reverse primers.

### 3.6. Phylogenetic Analysis

To evaluate Maurolipin coding sequence, researchers created a phylogenetic tree based on deposited protein-coding sequences in GenBank, including *Heterometrus fulvipes* (GenBank : DQ146998.1), *Mesobuthus tamulus* (GenBank : AY443497.1), *Pandinus cavimanus* (GenBank : JN315724.1), *Hadrurus spadix* (GenBank : GFAH01000435.1), *Mesobuthus gibbosus* (GenBank : KF770818.1), *Anuroctonus phaiodactylus* (GenBank : EF364043.1), *Hemiscorpius lepturus* (GenBank : KX924472.1), *Hemiscorpius lepturus* (GenBank : BK059885.1), *Opisthacanthus cayaporum* (GenBank : FM998793.1), *Scorpio maurus* (GenBank : MF347455.1), *Apis mellifera* (GenBank : EF373554.1), and *Homo sapiens* (GenBank : M86400.1) by utilizing the maximum likelihood method [34]. Multiple sequence alignment for evolutionary analyses was conducted in MEGA7 software based on the Clustal W method [35]. The percentage of replicate trees in which the associated taxa clustered together in the bootstrap test (1000 replicates) was shown next to the branches [36].

### 3.7. In Silico Analyses

#### 3.7.1. Structural Characteristics of Maurolipin

MEGA software (Version 7.0) was used for the sequence alignments. All primers were designed by the Gene Runner (Version 4) and Oligo 7 software. The designed primer specificity was determined using Primer-BLAST on NCBI (https://blast.ncbi.nlm.nih.gov/Blast.cgi). To identify the structural feature of Maurolipin and guarantee that the obtained sequence could be a part of phospholipase family proteins, Maurolipin coding sequence was translated by Gene Runner software (Version 4.0), and diverse instruments evaluated the concluded sequence. Protein BLAST (https://www.ncbi.nlm.nih.gov) was performed to identify proteins with great closeness to recognize and record their characteristics. To compare and classify protein structure, researchers chose similar proteins for alignment with Clustal Omega (https://www.ebi.ac.uk/Tools/msa/clustalo/). Amino acid compounds of Maurolipin were analyzed by utilizing ProtParam online tool (https://web.expasy.org/protparam/). To disulfide bridge prediction of the target protein, DiANNA 1.1 web server (https://clavius.bc.edu/∼clotelab/DiANNA/) was used [37, 38]. Disulfide bridge formation is essential for biological activity in many proteins [39].

#### 3.7.2. Three-Dimensional Structure Prediction

In order to predict the three-dimensional (3D) structure, the SWISS-MODEL online tool (https://swissmodel.expasy.org/) and Phyre2 (https://www.sbg.bio.ic.ac.uk/phyre2/html/page.cgi?id=indexfulvip) were used to predict the 3D structure of a query protein through the sequence alignment of template proteins. This model was chosen for superimposition. The predicted 3D structure was evaluated by UCSF Chimera software (Version 1.14).

#### 3.7.3. Active Site Structure

PLA_2 _breaks the sn-2 position of the glycerol backbone of phospholipids, mainly in a metal-dependent reaction, to produce lysophospholipid (LysoPL) and a free fatty acid (FA) [10, 11]. Superimposition of active site was carried out by UCSF Chimera (Version 1.14) and DeepView/Swiss-PdbViewer (Version 4.10) software. The root-mean-square deviation (RMSD) of bee venom PLA_2 _ and Maurolipin active-site residue was calculated by Chimera software (Version 1.14) to measure the average distance between corresponding atoms in two protein chains based on carbon alpha atoms. The active site prediction was undertaken by ExPASy-PROSITE (https://prosite.expasy.org/).

#### 3.7.4. Prediction of Protease Cleavage Sites

PROSPER (Protease Specificity Prediction Server) (https://prosper.erc.monash.edu.au/webserver.html) and PeptideCutter (https://web.expasy.org/peptide_cutter/) servers were used for prediction of the activated form of target protein.

## 4. Results

### 4.1. Characterization of Maurolipin Coding Sequence

**Figure 2:**
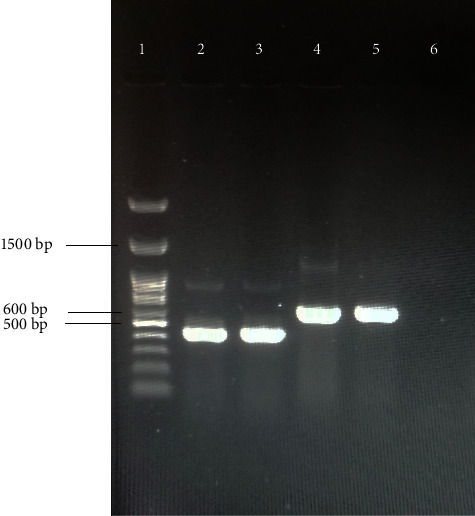
Colony PCR using specific PLA_2 _ and universal M13 primers. Lane 1 is ladder 100 (bp) DNA marker, Lanes 2 and 3 are PLA_2_ (432 bp) sequence amplified using specific primers, Lanes 4 and 5 are amplified gene using universal M13 primers (580 bp), and Lane 6 is negative control.

To identify Maurolipin coding sequence, RT-PCR was performed by specific primers on the synthesized complementary DNA (cDNA), which appeared as a fragment close to the expected size of 432 (bp) (Figure 1(d)). About colony PCR, the expected size of PCR product amplified using universal M13 primers was 580 bp (Figure 2). Maurolipin coding sequence was deposited in GenBank under accession number (MW241004).

### 4.2. Analysis of Maurolipin Coding Sequence

Maurolipin coding sequence contained a 432 bp open reading frame (ORF) that encoded a protein of 144 amino acid residues with a predicted molecular mass of 16.34 kDa. The amino acid sequence of Maurolipin was evaluated by Gene Runner software. The BLAST result showed that Maurolipin coding protein sequence was similar to Tunisian *S. maurus *PLA_2 _ (Sm-PLVG) (GenBank : MF347455.1) with 95.14% identity. Other listed proteins with high identity levels in BLAST search were 74.07% similar to Hemilipin from *H. lepturus* (GenBank : KX924472.1), 78.12% to PLA_2 _ from *O. cayaporum* (GenBank : FM998793.1), and 74.12% to PLA_2 _ from *A. phaiodactylus* (GenBank : EF364043.1). Maurolipin protein-coding sequence was assessed using protein BLAST alignment.

### 4.3. Phylogenetic Analysis

**Figure 3:**
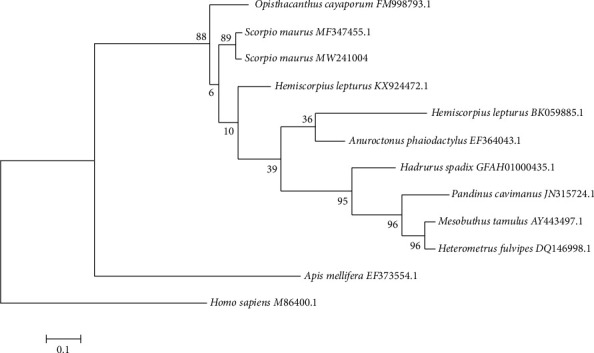
Phylogenetic relationship between different species of scorpions, *Apis mellifera*, and *Homo sapiens* based on PLA_2_ coding sequences. The accession number of each sequence has been shown in front of its name. The percentage of replicate trees in which the related taxa clustered together in the bootstrap test (1000 replicates) has been displayed next to the branches. The scale bar corresponds to 0.1 substitutions per nucleotide.

Initial tree(s) for the heuristic search were received automatically by applying Neighbor-Join and BioNJ algorithms to a matrix of pairwise distances estimated using the maximum composite likelihood (MCL) approach and then selecting the topology with higher level logarithm likelihood value. Instructive branch lengths were typically drawn to scale and showed the number of substitutions per site (0.1). The significant relationship between insect PLA_2 _ was quite evident. In addition, the phylogenetic analyses showed that the enzyme detected in humans differed significantly from that of insects (Figure 3).

### 4.4. In Silico Analyses

#### 4.4.1. Structural Characteristics of Maurolipin

**Figure 4:**
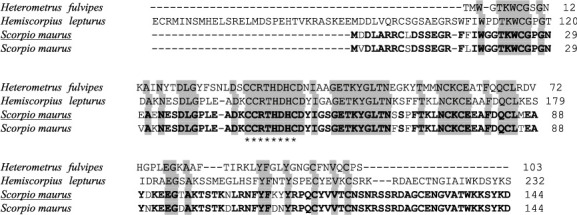
Sequence alignment of Maurolipin (Iranian *S. maurus*PLA_2_: underlined) with three reported PLA_2_ of other species of scorpions, including Tunisian *S. maurus* (GenBank : AVD99009.1), *Heterometrus fulvipes* (GenBank : Q3YAU5.1), and *Hemiscorpius lepturus* (GenBank : A0A1L4BJ46.1). Gaps are displayed by (-). Conserved amino acids among species are shaded in gray. Conserved amino acids between the same species of *S. maurus* are bolded. Conserved catalytic site is assigned by (^*∗*^).

**Figure 5:**
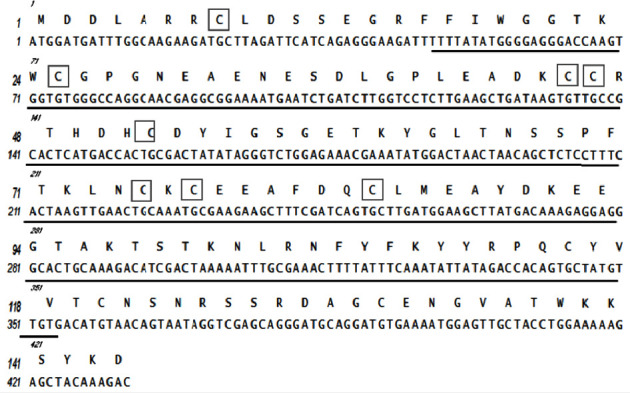
Nucleotides and predicted amino acid sequence of Maurolipin. Nucleotide and amino acid numbers appeared on the left. The middle-letter at above the nucleotide sequence is coded amino acid sequence. The disulfide bonds between cysteine residues in the protein structure have been signed by black boxes. The conserved domains of Maurolipin amino acid length with PLA_2_ superfamily have been shown by black line.

The consequences indicated that all selected proteins were associated with insect PLA_2 _. Above all, Maurolipin is high similar to Tunisian *S. maurus *PLA_2 _ (Sm-PLVG) (GenBank : AVD99009.1) with 90.28% identity. Sequence alignment of Maurolipin coding gene with three reported PLA_2 _ from different scorpion species including *S. maurus* (GenBank : AVD99009.1), *H. fulvipes* (GenBank : Q3YAU5.1), and *H. lepturus* (GenBank : A0A1L4BJ46.1) was performed by Clustal Omega and conserved amino acids were highlighted (Figure 4). The conserved amino acids between the two *S. maurus* species sequences of Maurolipin and Sm-PLVG (GenBank : AVD99009.1) were analyzed. Maurolipin did not resemble Sm-PLVG (GenBank : AVD99009.1) in 13 amino acids. The amino acid compounds of Maurolipin are shown in Table 1. The predicted disulfide bonds in five positions were between (Cys8 and Cys45), (Cys25 and Cys46), (Cys52 and Cys75), (Cys77 and Cys84), and (Cys115 and Cys131) (Figure 5).

#### 4.4.2. Three-Dimensional Structure Prediction

**Figure 6:**
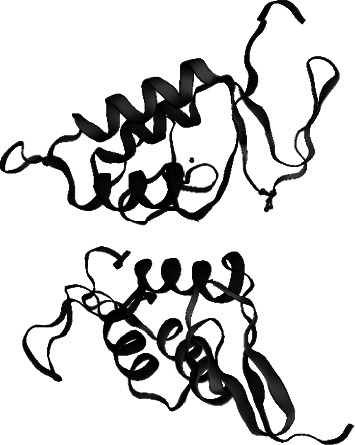
3D structure of PLA_2_ was predicted by SWISS-MODEL web server. Predicted 3D structure of Maurolipin with 38.83% identity to bee venom PLA_2_.

**Figure 7:**
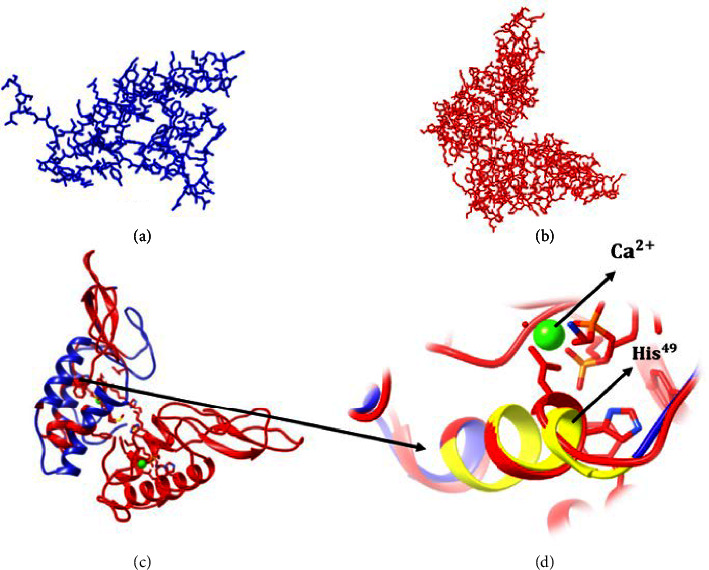
Superimposition of 3D structure of bee venom and Maurolipin. 3D structure of Maurolipin is similar to the chain A of bee venom PLA_2_. (a) and (b): the 3D structure predicted by DeepView/Swiss-PdbViewer (version 4.10) software. (c): the Superimposition predicted by UCSF Chimera (version 1.14) software. In both pictures, the blue color shows Maurolipin and the red color shows bee venom PLA_2_. (d): the yellow color shows Maurolipin active site.

The three-dimensional (3D) structure of Maurolipin by SWISS-MODEL demonstrated that Maurolipin was similar to chain A of bee venom PLA_2 _, group III subfamily, with 38.83% identity. The predicted 3D structure demonstrated that Maurolipin resembled chain A of bee venom PLA_2 _ (Figure 6). Superimposition of 3D structure of bee venom and *S. maurus *PLA_2 _ is shown in Figure 7. The 3D structure of Maurolipin by Phyre2 displayed that the target sequence was close to the insect PLA_2 _ family with 100% confidence and 39% identity. On the other hand, Maurolipin was similar to vertebrate PLA_2 _ with 97.3% confidence and 33% identity.

#### 4.4.3. Active Site Structure

The comparison of Maurolipin sequence with similarly characterized related proteins displayed a conserved catalytic site (active site), which was common in the PLA_2 _ superfamily of secretory and cytosolic enzymes. Conserved domains on Maurolipin were in the position of 17 to 118 amino acids length. According to the ExPASy-PROSITE online tool, the Maurolipin active site was in the position of 45 to 52 of amino acids length, which was conserved among different species of scorpions (Figure 4). The comparison of Maurolipin and bee venom active site was done by calculating the root mean square deviation (RMSD), which is reported in Table 2. The catalytic domain included the “CCRTHDXC motif” and the Ca^2+^ binding domain that supported the active site in the PLA_2 _ superfamily.

#### 4.4.4. Prediction of Protease Cleavage Sites

Based on PROSPER, three types of protease family cleavage Maurolipin coding sequences were determined and shown in Table 3. According to the PeptideCutter web server, the prediction enzymes including caspase-1 to −10, enterokinase, factor Xa, granzymeB, and thrombin cannot cut Maurolipin coding sequence.

## 5. Discussion and Conclusion

Prediction of protein structure is the focus of interest of many investigators. The current study was the first investigation of molecular characterization and in silico analyses of Maurolipin structure from venom glands of Iranian *S. maurus* that can be added to the literature when targeting molecular characterization of PLA_2 _ coding gene from venom glands of Iranian *S. maurus.* Phospholipase *A*_2_ (PLA_2 _) has a relevant role in of the inflammatory process, which catalyzes the hydrolyze phospholipids at the sn-2 position of the glycerol backbone and releases fatty acid and lysophospholipids [40, 41]. Group III subfamily of PLA_2 _ has been identified from various sources such as reptiles [42, 43], mammals [44], parasites [45], and arthropods including scorpion [20, 23]. The coding sequence of PLA_2 _ was detected from *S. maurus* venom glands for the first time in Iran. The detected PLA_2 _ has encoded a protein of 144 amino acid residue named Maurolipin. Till now, several studies reported the genes encoding phospholipases *A*_2_ from different species of scorpions, including Hemilipin from *H. lepturus* [23], Leptulipin from *H. lepturus* [25], Imperatoxin I and Phospholipin from *P. imperator* [21, 22], Phaiodactylipin from *A. phaiodactylus* [20], Heteromtoxin from *Heterometrus laoticus* [10], Phospholipase *A*_2_ from *H. fulvipes* [46], MtPL *A*_2_ from *Mesobuthus tumulus* [47], Sm-PLVG from Tunisian *S. maurus* [27], and Phospholipase *A*_2_ from *Hadrurus gertschi* [48].

The results of BLAST in NCBI showed that the detected sequence from the Iranian *Scorpio maurus* has a very high similarity to the same sequence of Tunisian *S. maurus* (GenBank : MF347455.1) [49]. However, this level of similarity is very low compared to other species of scorpions. Comparing the coding sequence of Maurolipin with other characterized PLA_2 _ from different species of scorpions showed that Maurolipin was a member of the PLA_2 _ superfamily.

Phylogenetically, there is a weak relationship between groups I, II, and III of phospholipase *A*_2_, but at calcium-binding site and the active site region, they are pretty similar [50, 51]. In the current study, the result of the likelihood analysis showed that the characterized PLA_2 _ from Iranian *S. maurus* and other arthropods, scorpions, and *Apis mellifera* (GenBank : FE373554.1) are well clustered. In contrast, the one from *Homo sapiens* (GenBank : M86400.1) is distantly located similar to the phylogenetic analyses of *A. phaiodactylus*PLA_2 _ [51].

The difference in 13 amino acids in the gene encoding sequence of Maurolipin and Sm-PLVG is most likely due to differences in the geographical condition in which they live, because the structure of a protein reflects its genetic sequence. The residues Asp^2^,  Leu^9^, Phe^17^, Glu^30^, Glu^32^, Ser^67^, Pro^69^, Met^86^, Asp^90^, Thr^95^, Asn^102^, Lys^109^, and Tyr^110^ of Maurolipin were Val, Ser, Leu, Val, Lys, Phe, Phe, Thr, Asn, Asp, Asp, Asp, and Asn in Sm-PLVG (GenBank : AVD99009.1), respectively. Indeed, the difference in the amino acid residue is more significant among different species of scorpions.

Based on the three-dimensional structure prediction results, Maurolipin is highly similar to the structure of phospholipase *A*_2_ that belonged to the group III subfamily; however, it was slightly similar to vertebrates' phospholipase *A*_2_. Phylogenetic analysis also confirms these results. Similar to characterized PLA_2 _ of *A. phaiodactylus* [51] and Imperatoxin I of *P. imperator* [21], Maurolipin is closely related to the genomic structure of *A. mellifera,* the only known representative structure in group III PLA_2 _. Evaluation of the 3D structure of the target gene revealed that the identified protein was very similar to the chain A of characterized PLA_2 _ from bee venom. Four disulfide bonds were predicted for phospholipase *A*_2_ of *M. tamulus* at the position of eight cysteines (Cys8-Cys30), (Cys29-Cys68), (Cys35-Cys61), and (Cys59-Cys96) [47]. It is similar to the position of the human group III PLA_2 _ disulfide bonds in ten cysteines (Cys8-Cys30), (Cys29-Cys68), (Cys35-Cys61), (Cys59-Cys91), and (Cys101-Cys113) while different positions were predicted to the five disulfide bonds in Maurolipin [52]. The histidine-aspartic (His-Asp) acid pair is stated necessary for the catalytic mechanism of the phospholipase *A*_2_. Active-site residues were universally conserved within protein families, displaying their key role for substrate catalysis [53]. In this study, RMSD of Maurolipin and bee venom PLA_2 _ was calculated. The RMSD value computes the average deviation between the equivalent atoms of two proteins depending on conformational differences and structural dimensions [54, 55]. The smaller the RMSD, the further similar the two structures. In this study, the active site residue of Maurolipin also has the His-Asp acid pair, and the effective amino acid in the catalytic domain was His^49^ while His^34^ played a significant role in the active site residue of bee's venom PLA_2 _ and Sm-PLVG [49].

## Figures and Tables

**Table 1:** Amino acid compounds of Maurolipin were analyzed by ProtParam online tool.

Amino acid type	Number	Percentage %
Glu (E)	12	8.3
Gly (G)	12	8.3
Lys (K)	12	8.3
Asp (D)	11	7.6
Ser (S)	11	7.6
Cys (C)	11	7.6
Thr (T)	10	6.9
Asn (N)	9	6.2
Ala (A)	8	5.6
Arg (R)	8	5.6
Leu (L)	8	5.6
Tyr (Y)	8	5.6
Phe (F)	6	4.2
Pro (P)	4	2.8
Trp (W)	3	2.1
Val (V)	3	2.1
Gln (Q)	2	1.4
His (H)	2	1.4
Ile (I)	2	1.4
Met (M)	2	1.4

**Table 2:** RMSD of active site residues in an important domain in Maurolipin and bee venom PLA_2_.

Amino acid	Cys	Cys	Arg	Thr	His	Asp	His	Cys
Position in Maurolipin	45	46	47	48	49	50	51	56
Amino acid	Cys	Cys	Arg	Thr	His	Asp	Met	Cys
Position in bee venom PLA_2_	30	31	32	33	34	35	36	37
RMSD	0.000	0.000	0.000	0.000	0.724	2.035	2.401	1.531

**Table 3:** Predicted cleavage sites of individual proteases. Restriction sites have been shown by (↓).

Protease family	Protease type	Position	Segment
Cysteine protease	Cathepsin K	56	DYIG↓SGET
		86	QCLM↓EAYD

Metalloprotease	Matrix metallopeptidase-9	63	TKYG↓LTNS
		84	FDQC↓LMEA
		102	STKN↓LRNF
		115	RPQC↓YVVT

Serine protease	Chymotrypsin A (cattle-type)	81	EEAF↓DQCL
		89	MEAY↓DKEE
		138	VATW↓KKSY
	Elastase-2	18	RFFI↓WGGT
	Cathepsin G	17	GRFF↓IWGG
		54	HCDY↓IGSG
		62	ETKY↓GLTN
		116	PQCY↓VVTC
		121	VTCN↓SNRS
	Glutamyl peptidase I	41	GPLE↓ADKC
		79	KCEE↓AFDQ
		132	AGCE↓NGVA
	Thylakoidal processing peptidase	52	HDHC↓DYIG

## Data Availability

All data generated or analyzed during this study are available upon reasonable request to the corresponding author.

## Abbreviations

PLA_2 _: Phospholipase A_2_
cPLA_2 _: Cytosolic PLA_2 _
PAF-AH: Platelet-activating factor cetyl hydrolases
iPLA_2 _: *Ca* ^2+^−Independent PLA_2 _
AdPLA_2 _: Adipose-specific PLA_2 _
sPLA_2 _: Secreted phospholipase A_2_
OA: Oleic acid
AA: Arachidonic acid
cDNA: Complementary DNA
PCR: Polymerase chain reaction
bp: Base pair
kDa: Kilodaltons
ORF: Open reading frame
X-Gal: 5-Bromo-4-chloro-3-indolyl-beta-D-galactopyranoside
IPTG: Isopropyl *ß*-D-1-thiogalactopyranoside
LB: Luria-Bertani
MCL: Maximum composite likelihood
3D: Three-dimensional structure
NCBI: National Center for Biotechnology Information
RT-PCR: Reverse Transcription Polymerase Chain Reaction
μL: Microliter
Met: Methionine
TBE: Tris borate EDTA
GSPs: Gene-specific primers
RMSD: Root-mean-square deviation
Thr: Threonine
Asn: Asparagine
Lys: Lysine
Tyr: Tyrosine
Asp: Aspartic acid
Cys: Cysteines
His: Histidine
Val: Valine
Leu: Leucine
Phe: Phenylalanine
Glu: Glutamic acid
Ser: Serine
Pro: Proline.
